# Silver Core Coated with Molecularly Imprinted Polymer as Adsorbent in Pipet-Tip Solid Phase Extraction for Neonicotinoids Determination from Coconut Water

**DOI:** 10.1021/acsmeasuresciau.4c00036

**Published:** 2024-08-28

**Authors:** Laíse Aparecida Fonseca Dinali, Anny Talita Maria da Silva, Keyller Bastos Borges

**Affiliations:** Departamento de Ciências Naturais, Universidade Federal de São João del-Rei (UFSJ), Campus Dom Bosco, Praça Dom Helvécio 74, Fábricas, 36301-160 São João del-Rei, Minas Gerais, Brazil

**Keywords:** silver nanoparticles, pesticides, sample preparation, core−shell, molecularly imprinted polymer, HPLC

## Abstract

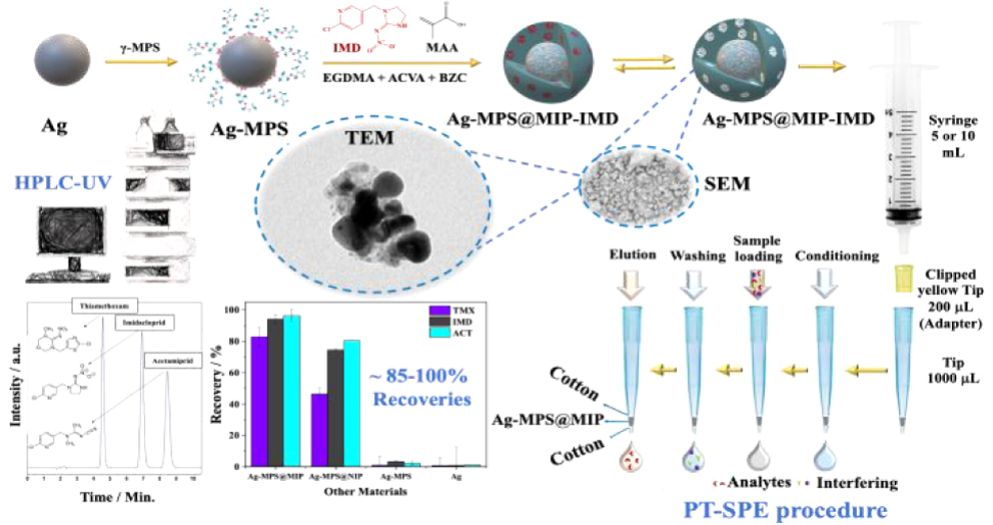

In this work, we report an innovative adsorbent named Ag-MPS@MIP that has a core@shell structure, i.e., silver nanoparticles modified with 3-methacryloxypropyltrimethoxysilane as the core and molecularly imprinted polymer based on methacrylic acid as its shell. Thiamethoxam, imidacloprid, and acetamiprid were extracted from coconut water samples using Ag-MPS@MIP in pipet-tip solid phase, prior to high-performance liquid chromatography analysis. The separation was carried out on isocratic mode using a mobile phase consisting of C18 column (Phenomenex, 150 mm × 4.6 mm, 5 μm), ultrapure water acidified with 0.3% phosphoric acid:acetonitrile (78:22, v/v), flow rate at 1.0 mL min^–1^, injection volume of 10 μL, temperature of 25 °C, and wavelength at 260 nm. The adsorbent and precursor materials were properly characterized by different instrumental techniques. The main factors affecting the recovery of analytes from coconut water samples by pipet-tip solid phase were optimized, such as sample volume (250 μL), sample pH (pH = 5.0), ionic strength (1%, m/v), washing solvent (300 μL ultrapure water), volume and type of eluent (500 μL methanol), amount of adsorbent (15 mg), cycle of percolation–dispensing (1×), and reuse (5×). Thereby, the neonicotinoids presented extraction recoveries between 82.80 and 96.36%, enrichment factor of 5, linearity ranged from 15 to 4000 ng mL^–1^, correlation coefficient (*r*) > 0.99, limit of detection of 5 ng mL^–1^, satisfactory selectivity, stability, and proper precision (RSD%: 0.52–9.64%) and accuracy (RE%: −5.19–6.45%). The method was successfully applied to real samples of coconut water.

## 1 Introduction

Neonicotinoids, which are systemic pesticides derived from nicotine, were developed in the 1970s by Shell Development Company (Modesto, CA, USA). The first neonicotinoid compound developed was nithiazine, a compound that, like nicotine, maintained nicotinic acetylcholine receptors but with significant toxicity only to insects. This chemical was not effective in field use due to its low photostability, and as a result, it was not marketed.^1^ It was only at the beginning of the 1990s that Nihon Bayer Agrochem (Japan) launched imidacloprid (IMD, Figure S1), the first neonicotinoid with selective insecticidal action on insects, adequate photostability, and low toxicity in mammals, justifying its commercial viability. IMD and acetamiprid (ACT, Figure S1) are classified as first-generation neonicotinoids.^1^ The main representative of the second generation is thiamethoxam (TMX, Figure S1), which was launched on the market in 1998 with the aim of surpassing the effectiveness of first-generation compounds against different types of insects, with emphasis on great systemic activity, greater residual effect, and stability.^2^ More than 30 years after the commercialization of the first neonicotinoid active, this class has become the most used worldwide.^3^ The systemic action and versatility of application of these pesticides, including prophylactically in the soil and seeds, contributed to their prominence in the agrochemicals market.^4^

Although the growing global demand for food consumption is a reality, it is necessary that large-scale production does not violate the population’s principles of food security. For this, it is necessary that the degree of exposure to pesticide residues is constantly monitored.^5^ Thus, it is crucial to create new analytical and technical approaches that permit more control over the use of pesticides within the limits set by law as well as the effects on the environment and public health.

It is vital to complete the sample preparation step, which tries to reduce the presence of interferents and simultaneously extract the analytes in order to identify and quantify these pesticides in food samples. In this context, pipet-tip solid phase extraction (PT-SPE) is a miniaturization of solid phase extraction (SPE) that uses a micropipet tip instead of the traditional extraction cartridge. Smaller amounts of adsorbent, sample, and solvent are required.^6^ PT-SPE was used for the extraction of pesticides in different matrices employing varied adsorbents, such as graphene for extraction of carbamates from fruit juice,^7^ and zirconium-based metal–organic frameworks (Zr-MOFs) for determination of herbicides in cucumber samples, soil, and water.^8^ The use of selective adsorbents with different approaches has also been reported, such as the extraction of atrazine and its degradation products from Chinese yam; for screening organochlorines in spinach; and for colorimetric determination of a pyrethroid metabolite in water samples.^9−11^ There were no studies that employed selective adsorbents in conjunction with PT-SPE to extract neonicotinoids from any kind of matrix.

Molecularly imprinted polymers (MIP) are adsorbents produced through molecular imprinting technology, thus exhibiting the property of binding and rebinding specifically to a given target molecule. In addition to having high selectivity, they have high physical and chemical stability, a broad spectrum of applications, well-established syntheses in the scientific community, and a relatively high ability to improve their final structure, for example, through surface imprinting technique.^12^ This strategy contributes to the molecular recognition sites being located on the surface of the particles, which reduces resistance to mass transfer, favoring adsorption kinetics due to greater binding capacity and greater surface area, especially when compared to MIP that are not core–shell.^13^

Generally, in materials with a core–shell structure, the particle serving as the nucleus not only supports the shell but also contributes stability and additional functions to the final material in a variety of applications, including extraction methods,^13^ chromatographic separation,^14^ sensors,^15^ and controlled drug delivery.^16^ Although silica and materials with magnetic properties have been widely used as a core, other materials have gained prominence, such as quantum dots, carbon dots, MOFs, graphene, and noble metal nanoparticles (silver and platinum).^17^ Silver (Ag) has been used in the composition of nanocomposites for application in SPE techniques, while its use in MIP-based core–shell composites has been reported mainly for applications in sensors, controlled drug release, and surface-enhanced Raman scattering.^18−20^ To date, no studies have been reported using a silver-based core–shell composite and MIP in sample preparation techniques.

In this work, we report the innovative development of an adsorbent with a core@shell structure that has silver nanoparticles modified with 3-methacryloxypropyltrimethoxysilane (γ-MPS) as a core and an MIP based on methacrylic acid (Ag-MPS@MIP) as its shell. This material, after being properly synthesized and characterized by thermogravimetric analysis, Fourier transform infrared spectroscopy, X-ray diffraction, pH of point zero charge, textural properties (specific surface area, volume, and pore diameter), scanning electron microscopy with energy-dispersive spectrometry analysis, transmission electron microscopy, and wettability, has been used as an adsorbent in PT-SPE for the simultaneous and selective determination of neonicotinoids from coconut water samples. All parameters that affect sample preparation were evaluated and discussed in detail. We address also the entire optimization, validation, and application of method. Furthermore, the material developed in this work has unprecedented application and can potentially contribute to applications that require antimicrobial properties, such as the development of packaging and for the determination of neonicotinoid residues in different matrices.

## 2 Experimental Section

### 2.1 Reagents and Solvents

Reagents and solvents with a high degree of purity were used in all stages of development of this study, as well as in the synthesis of materials, sample preparation, and chromatographic analysis. Thus, silver nitrate (AgNO_3_) and phosphoric acid were purchased from Neon (Suzano, SP, Brazil), citric acid and hydrochloric acid from Vetec (Duque de Caxias, RJ, Brazil), ascorbic acid and sodium hydroxide from Synth (Diadema, SP, Brazil), ethylene glycol dimethacrylate (EGDMA), methacrylic acid (MAA), benzalkonium chloride (BZK), γ-MPS and methanol (high-performance liquid chromatography (HPLC) grade) from Sigma-Aldrich (St. Louis, MO, USA), 4,4′-azobis (4-cyanovaleric acid) (ACVA) from Santa Cruz Biotechnology (Dallas, TX, USA), sodium chloride from Qhemis High Purity (Jundiaí, SP, Brazil), acetic acid and ethyl alcohol from Êxodo (Sumaré, SP, Brazil), acetonitrile and isopropanol (both HPLC grade from J. T. Baker (Mexico City, Mexico)), and chloroform and dichloromethane from Dinâmica (Diadema, SP, Brazil). The water was distilled and purified with a Millipore Milli-Q Plus system (Bedford, MA, USA).

### 2.2 Standard Solution

Neonicotinoids standards were purchased from the United States Pharmacopeia Reference Standard: TMX (99.5%, w/w), IMD (98.10%, w/w), and ACT (99.5%, w/w). The neonicotinoid standard solutions were prepared in methanol, at a concentration of 1 mg mL^–1^, which were stored under photoprotection and refrigeration at −20 °C. These solutions were used throughout the study to prepare solutions at 10 μg mL^–1^ and in the concentration range of 15–4000 ng mL^–1^, for application in sample preparation and for construction of the analytical curve, respectively.

### 2.3 Instrumentation

The chromatographic separations were performed on an HPLC Agilent equipped with a quaternary pump (1260 G1311B), thermostat (1260 G1330B), automatic injector (1260 Hip ALS G1367E), column oven (1290 TCC G1316C), ultraviolet/visible detector (1260 VWD G1314F), and Agilent OpenLAB Chromatography Data System data acquisition system. The separations were carried out in isocratic mode using a mobile phase consisting of Phenomenex C18 column (150 mm × 4.6 mm, 5 μm), ultrapure water acidified with 0.3% phosphoric acid:acetonitrile (78:22, v/v), flow rate at 1.0 mL min^–1^, injection volume of 10 μL, temperature at 25 °C, and wavelength at 260 nm. After optimizing the method, system suitability experiments were carried out, such as retention time (*T*_R_), retention factor (*k*), separation factor (α), number of theoretical plates (*N*), resolution (*R*_S_), and asymmetry factor (*A*_f_).

### 2.4 Synthesis and Functionalization of Silver Nanoparticles

In a beaker containing 100 mL of distilled water, 10 mL of 1.0 mol L^–1^ AgNO_3_ aqueous solution and 1 mL of 0.25 mol L^–1^ citric acid solution were added, under moderate stirring and in an ice bath. Then, under vigorous stirring, 10 mL of a 1 mol L^–1^ aqueous ascorbic acid solution was quickly added. The solution turned gray, and the reaction continued for 15 min. The Ag precipitate was separated by centrifugation, washed a few times with distilled water, and dried in an oven at 60 °C for 24 h.^21^

The modification of silver nanoparticles was carried out according to the literature with some modifications.^22^ Briefly, 500 mg of Ag nanoparticles was added to a flat-bottom flask, which was dispersed in 40 mL of ethanol:ultrapure water solution (4:1, v/v). Next, 8 mL of γ-MPS was added. The mixture was subjected to ultrasound for 10 min and then magnetically stirred at 40 °C for 24 h. After this period, the functionalized Ag nanoparticles (Ag-MPS) were washed several times with ethanol and then dried in an oven at 60 °C for 24 h.

### 2.5 Synthesis of MIP and NIP Core@Shell

In an amber vial, 0.4 mmol of template (IMD) and 2 mmol of functional monomer (MAA) were dissolved in 20 mL of acetonitrile (porogenic solvent). This solution was subjected to an ultrasonic bath for 1 h, taking into account the prepolymerization phase, which is when the template-monomer complex forms. Meanwhile, in a beaker, 500 mg of the Ag-MPS was dispersed in 20 mL of acetonitrile for 1 h in an ultrasonic bath. Then, this suspension was transferred to a flask containing the template-monomer complex, together with 12 mmol of cross-linker (EGDMA), 80 mg of the radical initiator (ACVA), and 0.4 mmol of surfactant (BZK). The reaction medium was degassed for 30 min in ultrasound at room temperature (23 ± 3 °C), and then the flask was properly sealed and subjected to mechanical agitation at 75 °C for 24 h.

The template was completely removed from the Ag-MPS@MIP structure by successive washing with a solution of methanol:acetic acid (9:1, v/v) and then with acetonitrile. The removal of IMD was monitored by HPLC-UV analysis of the washing solution. The nonimprinted polymer (Ag-MPS@NIP) was synthesized under the same conditions, but in the absence of IMD.

### 2.6 Characterizations

Morphology and microstructural characteristics were observed by scanning electron microscopy (SEM) and transmission electron microscopy (TEM) using a TM3000 Hitachi Analytical Table Top microscope with acceleration voltage varying between 5 and 15 kV and a JEOL brand microscope, model JEM1400, operating with acceleration voltage up to 120 kV and 0.3 nm resolution, respectively. The SEM microscope equipped with an energy-dispersive spectroscopy (EDS) detector allowed semiquantitative analysis of the elementary chemical composition of the materials. The thermal stability and the content of inorganic and organic charge present in the adsorbents were evaluated by thermogravimetric analysis (TGA), using a 2950 TA Instruments thermobalance heated from 25 to 1000 °C with a heating rate of 10 °C min^–1^ and under nitrogen flow (50 mL min^–1^). The presence of the main functional groups was evaluated by Fourier transform infrared (FTIR) spectroscopy using an Agilent Technologies spectrometer, model Cary 630 FTIR with ATR module, spectral range from 4000 to 400 cm^–1^, 16 scans, and 4 cm^–1^ resolution. Nitrogen adsorption/desorption data were collected by a Quantachrome Autosorb-iQ2 Station 2, and Brunauer–Emmett–Teller (BET) and Barrett–Joyner–Halenda (BJH) analyses were performed to determine specific surface area, volume, and pore diameter. X-ray diffraction (XRD) was carried out on a Shimadzu model XRD 6100 X-ray diffractometer with copper radiation source Kα1 = 1.54059 Å and Kα2 = 1.54443 Å, voltage of 30 kV, and scan speed of 2 degrees min^–1^ in the range 2θ = 10 to 80°. The average crystallite size was estimated using the Scherrer equation (eq 1), which uses experimental data from the full width at half-maximum (FWHM) of diffraction peaks^23^
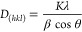
1where *D* is the size of the crystallite in the *hkl* direction, *K* is a dimensionless constant related to the shape of the crystallite; in this case, the value of 0.9 was used, considering the spherical shape of the particle; λ is the wavelength of the radiation source (Cu kα1 = 0.15406 nm); β refers to the FWHM value in radians; and θ corresponds to the Bragg angle.

The pH of point of zero charge (pH_PZC_) was determined using a Mettler Toledo FiveEasy pH/mV bench meter (Columbus, OH, USA). pH_PZC_ was experimentally determined by the pH shift method, using NaCl solution 0.001 mol L^–1^ as a supporting electrolyte solution.^24^ Thus, the initial pH of the support solution was adjusted with NaOH and HCl, both at a concentration of 0.1 mol L^–1^, producing solutions with pH 2.0, 4.0, 6.0, 8.0, 10.0, and 12.0. Then, 5 mL of each solution was added (*n* = 2) to Falcon tubes containing 12.5 mg of adsorbent. The tubes were placed on a shaking table at 2000 rpm for 15 min, at room temperature (25 ± 3 °C), and subsequently left to rest for 24 h. After this period, the final pH of each solution was measured. pH_PZC_ was defined by the point of intersection of pH_initial_ × pH_final_. This procedure was carried out under the same conditions as those for Ag-MPS@MIP and Ag-MPS@NIP.

Wettability was evaluated based on the contact angle (θ) formed by the interaction of a drop of water with the surface of each synthesized material. For this, high-resolution images generated from a Nikon D90 camera equipped with a Nikon 50 mm lens were taken.

### 2.7 PT-SPE Procedure

Coconut water sample, free of contaminants, was purchased from a local supermarket in São João del-Rei, Minas Gerais, Brazil. The sample was subjected to simple filtration to remove suspended particles using a Millipore Millex-GV hydrophilic poly(vinylidene fluoride) (PVDF) 0.45 μm filter. Then, 300 mL of sample was stored in a freezer, at a temperature of −20 °C, to be used in the optimization, Ag-MPS@MIP selectivity study, and validation of the analytical method.

Sample preparation was carried out using coconut water samples enriched with neonicotinoids at a concentration of 10 μg mL^–1^, and several parameters that directly affect the extraction efficiency/recovery of PT-SPE were optimized so that the best extraction conditions were achieved, such as the influence of the type and volume of the elution solvent, the volume and pH of the sample, the amount of adsorbent, the type of washing solvent, the salting-out effect (ionic strength), and the number of cycles of percolation–dispensing. To optimize the sample preparation, the following initial conditions were used: 5 mg of Ag-MPS@MIP, 500 μL of sample at pH 5.0 without added salt, with one percolation–dispensing cycle, 500 μL of ultrapure water as washing solvent, and 500 μL of acetonitrile as eluent. The adsorbent was then conditioned with 300 μL of methanol and 300 μL of ultrapure water.

After optimization of all previously mentioned parameters, the reuse capacity of the device and the comparison of recoveries of Ag-MPS@MIP with Ag-MPS@MIP, Ag-MPS, and Ag were performed.

### 2.8 Imprinting Effect Test for Ag@MIP

Distribution coefficient (*K*_d_) (eq 2), selectivity coefficient (*K*) (eq 3), and relative selectivity coefficient (*K′*) (eq 4) were used as selectivity parameters to measure the molecular recognition capacity of Ag-MPS@MIP against other molecules. The selectivity of Ag-MPS@MIP for TMX and ACT was also evaluated using Ag-MPS@MIP, for which IMD was used as a template. To this end, adsorption studies were carried out using 15 mg of adsorbent, 250 μL of coconut water sample at pH 5.0 enriched with neonicotinoids, and also other pesticides, including pyriproxyfen, deltamethrin, etofenprox, fipronil, fluazuron, azamethiphos, and chlorpyrifos (Figure S2), all at a concentration of 10 μg mL^–1^.
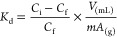
2where *C*_i_ and *C*_f_ represent the initial and final concentrations of the analytes in solution, respectively, *V* is the volume of the solution, and *mA* is the mass of the adsorbent material.
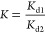
3where *K*_d1_ is the *K*_d_ obtained for the template and *K*_d2_ is the *K*_d_ of the interferents.
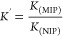
4

### 2.9 Method Validation

Selectivity was evaluated by comparing the chromatographic analysis of blank matrix, i.e., without the presence of the pesticides, and the coconut samples enriched with neonicotinoid standards at a concentration of 1 μg mL^–1^.^25^ The limit of detection (LOD) was determined experimentally (*n* = 5) from the lowest concentration detectable by the analytical technique. The limit of quantification (LOQ) was defined based on the lowest concentration of the analytes that can be measured with adequate precision and accuracy, i.e., relative standard deviation (RSD%) and relative error (RE%) values below 20% (*n* = 5).^25^ Linearity was verified in the range of 15–4000 ng mL^–1^ covering six independent concentrations (*n* = 3). The linear working range was defined through statistical treatment using the linear regression equation estimated by the ordinary least-squares method, together with the linear correlation coefficient (*r*). An analysis of variance (ANOVA) of the regression was also performed using the *F* test for lack of fit to verify the impact of the concentration of each point on the curve on the corresponding analytical response.^25^ The precision of the method was assessed by means of repeatability and intermediate precision using the concentrations 800, 2400, and 4000 ng mL^–1^ with five replicates for each point. The acceptance criterion was defined as the RSD% of the determinations (*n* = 5) being less than 15%.^25,26^ Accuracy was determined by means RE% with values less than 15% being accepted.^25,26^ Stability was checked on samples at a concentration of 800 and 4000 ng mL^–1^ subjected to freezing for 96 h to check long-term stability and resting for 24 h on the bench at room temperature (23 ± 3 °C) to assess short-term stability. Stability was assessed using the RSD% of each level studied and also by comparison with freshly prepared solutions. The analytical responses obtained for solutions under the studied stability conditions and for fresh solutions were statistically treated by Student’s *t* test with a significance level of 5% (*p* < 0.05).^25,26^

### 2.10 Method Application

Different samples of coconut water were used, without certification as an organic product, purchased in markets in the city of São João del-Rei, MG, Brazil. The samples were filtered through a 0.45 μm Millipore Millex-GV hydrophilic PVDF filter to remove suspended particles, and then the pH was adjusted to 5.0 and the PT-SPE procedure was carried out. The eluates were dried, resuspended in 50 μL of methanol, and submitted to chromatographic analysis.

## 3 Results and Discussion

### 3.1 Synthesis and Characterization of the Materials

**Figure 1:**
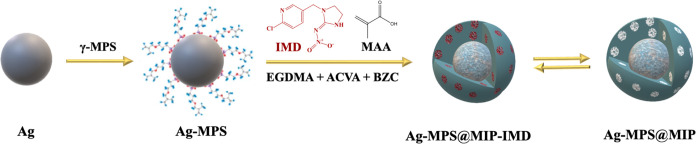
Schematic representation of Ag-MPS@MIP preparation.

The synthesis of Ag-MPS@MIP took place in three stages, as shown in Figure 1. First, silver nanoparticles were produced by the direct reduction of Ag^+^ ions with ascorbic acid, using AgNO_3(aq)_ as the metal precursor. Citric acid was used as a stabilizing agent, as it tends to encourage the formation of spherical silver particles.^21^ To make it possible to synthesize MIP over silver nanoparticles, they had to be functionalized with γ-MPS. In the synthesis of Ag-MPS@MIP, in addition to the reagents traditionally used, the cationic surfactant BZK was added, as previous studies by our research group have shown that this favors the formation of material with a larger surface area and pore diameter.^27^ Silver nanoparticles were chosen due to their binding properties, as well as their ease of synthesis, in which particle size, morphology, structure, and size distribution can be easily controlled by the synthesis concentration of the precursor. The large surface area of silver nanoparticles increases high surface energy, which can promote surface reactivity and sorption in the adsorptive sites.^18−20^

#### 3.1.1 Thermogravimetric Analysis

**Figure 2:**
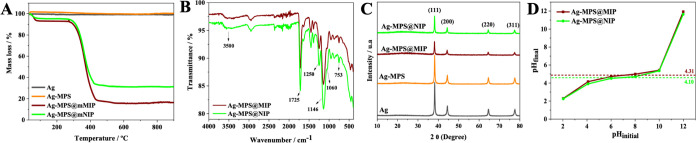
(A) Thermogravimetric analysis; (B) Fourier transform infrared spectroscopy; (C) X-ray diffraction; and (D) pH of point zero charge of the synthesized materials: Ag, Ag-MPS, Ag-MPS@MIP, and Ag-MPS@NIP.

Figure 2A shows that pure silver showed insignificant mass loss within the temperature range studied, from 10 to 1000 °C. In addition, the mass loss profiles of Ag-MPS@MIP and Ag-MPS@NIP were quite similar. The Ag-MPS curve showed a very similar profile to Ag nanoparticles, although the functionalized silver showed a mass loss of 2.04%, precisely due to the presence of γ-MPS. Both had small mass loss event at the beginning of the analysis of 6.19 and 3.95% respectively, due to humidity and possible remnants of volatile compounds that did not react during synthesis. It can be seen in the thermogram that at 351 °C there was a rapid and marked loss of mass corresponding to the decomposition of the polymeric part of the samples. This means that Ag-MPS@MIP and Ag-MPS@NIP had 75.39 and 63.45% of the polymer phase in their composition, respectively. There is also a slight loss of mass in the curves of both materials, around 2.43% for MIP and 1.24% for NIP, due to degradation products formed during thermal decomposition. At the end of the analysis, 16.40% by mass remained for Ag-MPS@MIP and 31.45% for Ag-MPS@NIP, which correspond to the inorganic charge present. These results suggest that the polymer phase of Ag-MPS@MIP was slightly larger than that of Ag-MPS@NIP. The molecular imprinting process, as well as the presence of the mold-monomer complex during polymerization, may have contributed to this difference in composition.

#### 3.1.2 Fourier Transform Infrared Spectroscopy

As shown in Figure 2B, the broad peak at approximately 3500 cm^–1^ refers to the stretching of the O–H bonds of the carboxyl, confirmed by the presence of an intense peak at 1725 cm^–1^ due to the stretching of the C=O bond of the carbonyl of the carboxylic acid present in the MAA. EGDMA contributed to the formation of an intense peak at 1146 cm^–1^ due to axial deformation of the C–O ester bond, along with a moderate intensity peak at 1250 cm^–1^ related to asymmetric stretching of the C–O bond. The peak at approximately 1060 cm^–1^, almost in the same absorption region of the C–O bond, is related to the asymmetric stretching of the Si–O–Si bond, which together with the peak at approximately 753 cm^–1^, related to bending vibrations of Si–O, is attributed to γ-MPS. Other absorption peaks observed at 1633, 1455, 1390, 2995, and 2953 cm^–1^ can be attributed, respectively, to the vibration of the remaining C=C bond of the cross-linker, to the angular deformation from the CH_2_–CH_2_ bond, to the symmetric bending vibration of the methyl groups, and the C–H stretching of the CH_2_ and CH_3_ groups.^28,29^

#### 3.1.3 X-ray Diffraction

Figure 2C shows that silver had four characteristic diffraction peaks in the range 2θ = 10–80° located at 38.15, 44.34, 64.50, and 77.47°, which can be attributed to the respective crystal planes (111), (200), (220), and (311) of the face-centered cubic structure.^22^ These results are in agreement with the silver diffraction pattern available in the American Mineralogist Crystal Structure Database and show that the nanoparticles are metallic silver (Ag^0^) since the diffraction peaks characteristic of silver oxide (Ag_2_O) were absent.^28,29^ The same crystallinity can be seen in the other synthesized materials, although with less intense peaks due to the coating steps. In addition, amorphous scattering was formed in the approximate range 2θ = 16–32° of lower intensity in the Ag-MPS diffraction curve and higher intensity in the Ag-MPS@MIP and Ag-MPS@NIP curves, referring to the formation of the amorphous phase. The average crystallite size/standard deviation estimated from the FWHM of the diffraction peaks corresponding to the crystal plane (111) was 20.90 ± 0.91 nm. Thus, it is possible to say that the proposed synthesis method led to the formation of silver nanoparticles that maintained their crystalline structure after coating with the polymeric phase.

#### 3.1.4 pH of Point Zero Charge

The pH of point zero charge provides important information regarding the surface charges of the material in relation to the pH of the medium. Figure 2D shows the ratio of the experimental curves obtained in this test for pH_final_ × pH_initial_. Ag-MPS@MIP had a value of 4.31, which means that at this pH, its surface electrical charge is neutral. In solutions with a pH lower than 4.31, the surface charges will be predominantly positive, which will favor the adsorption of anionic species from the medium. In solutions with a pH greater than 4.31, the adsorbent will be negatively charged and will be more powerful at adsorbing cations. The pH of the point zero charge of Ag-MPS@NIP was slightly lower, possibly due to the change in the chemical environment caused by the template during the synthesis of Ag-MPS@MIP.

#### 3.1.5 Textural Properties

Ag-MPS@MIP had a high surface area and pore size close to the maximum limit for micropores since the value obtained was very close to 2 nm (Table 1). Thus, it is possible that this adsorbent is made up mostly of micropores, but it is possible that there are mesoporous fractions.

**Table 1:** Specific Surface Area, Volume, and Pore Diameter Determined by BET and BJH Equations

parameter	Ag@MIP
specific surface area (m^2^ g^–1^)	120.09
pore volume (cm^3^ g^–1^)	0.09
pore size (nm)	1.83

#### 3.1.6 Scanning Electron Microscopy with Energy-Dispersive Spectrometer Analysis

**Figure 3:**
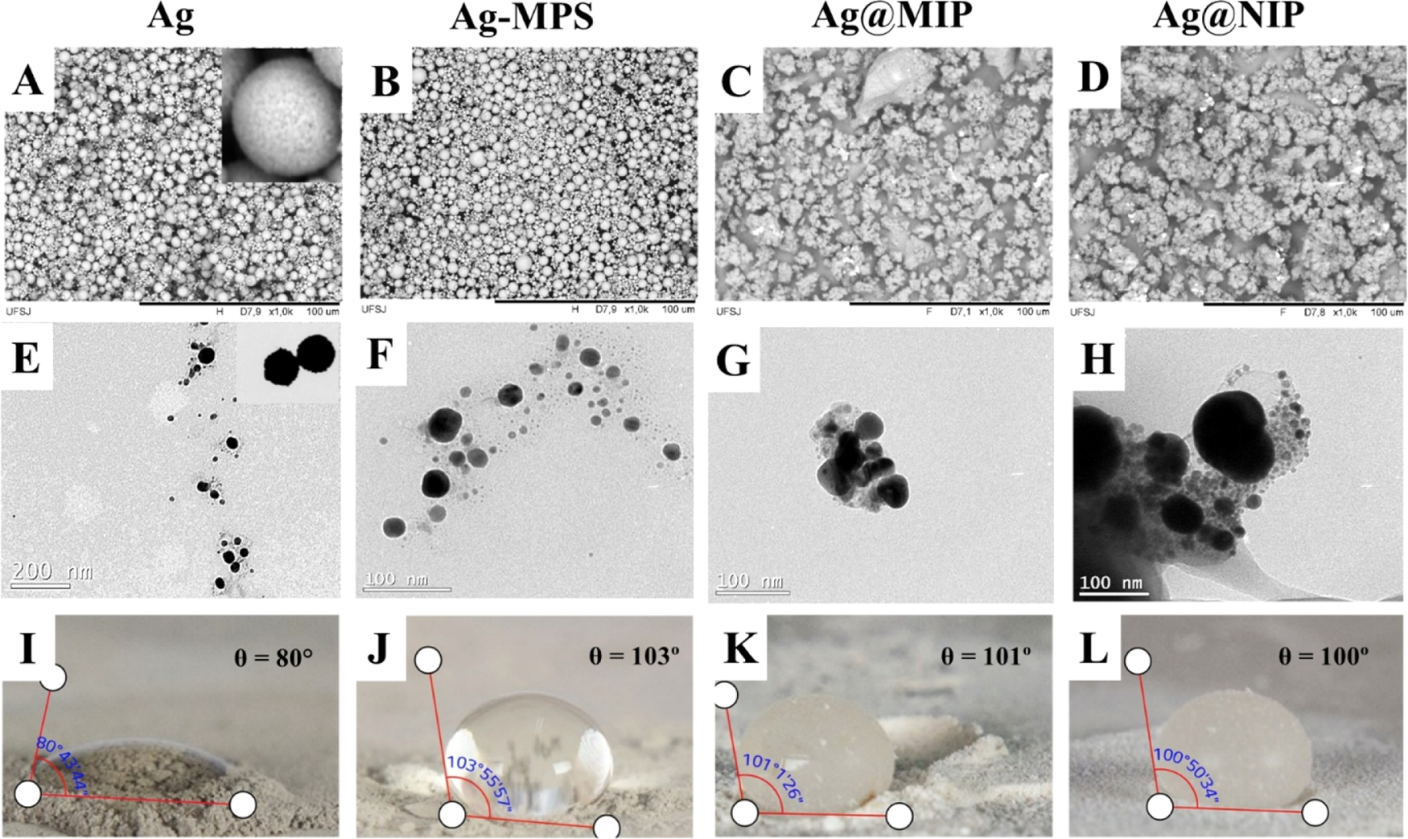
Scanning electron microscopy at a magnification of 1000× (A–D); transmission electron microscopy (E–H); and wettability (θ) (I–L) for Ag, Ag-MPS, Ag-MPS@MIP, and Ag-MPS@NIP, respectively.

Figure 3A,B shows the micrographs obtained for silver before and after functionalization with γ-MPS. It was observed that they were very similar; both had a spherical shape and irregular size distribution. The images of Ag-MPS@MIP and Ag-MPS@NIP are shown in Figure 3C,D. It is noted that there was an aggregation between the silver nanoparticles and the polymer, resulting in a well-dispersed material, with a heterogeneous and irregular surface, in addition to an uneven size distribution. These characteristics are desirable as they can contribute to greater adsorbent capacity.^30^

Table S1 shows the results of the semiquantitative elemental analysis of the materials. They all have silver in their composition, although in smaller quantities in Ag-MPS@MIP and Ag-MPS@NIP due to the polymeric coating. The presence of silicon in all materials, with the exception of silver, can be attributed to the γ-MPS functionalization reagent, which also contributed to the presence of carbon and oxygen in Ag-MPS. Carbon and oxygen were also detected in high percentages in Ag-MPS@MIP and Ag-MPS@NIP, which came from the reagents used in the synthesis, mainly MAA and EGDMA. This analysis helped to demonstrate that the polymerization process over the surface of silver nanoparticles was efficient.

#### 3.1.7 Transmission Electron Microscopy

Figure 3E shows a micrograph of silver, which presents well-dispersed particles and a clear spherical shape. Figure 3F shows the micrograph of Ag-MPS, the image of which shows the presence of a second phase that can be related to the coating with the functionalization reagent. The images for Ag-MPS@MIP and Ag-MPS@NIP can be seen in Figure 3G,H. Both micrographs suggest that the silver nanoparticles, seen in the images as the darker part, were surrounded by the polymer layer during synthesis. This means that the core–shell structure was formed and that the polymer adhered to the silver core.

#### 3.1.8 Wettability

The images from this essay are presented in Figure 3I–L. Pure silver formed a contact angle of 80° with the water droplet, exhibiting a hydrophilic character; however, after functionalization, the angle increased to 103°, which suggests that the γ-MPS reagent actually modified its surface, making it hydrophobic. After polymerization, the hydrophobic character was also observed for both Ag-MPS@MIP and Ag-MPS@NIP, which exhibited similar angles, 101 and 100°, respectively. This information indicates that the synthesized adsorbents are nonpolar in nature, which is consistent with most polymeric adsorbents. This may contribute to the adsorption of analytes with low polarity present in aqueous matrices.

### 3.2 PT-SPE Optimization

#### 3.2.1 Elution Solvent

**Figure 4:**
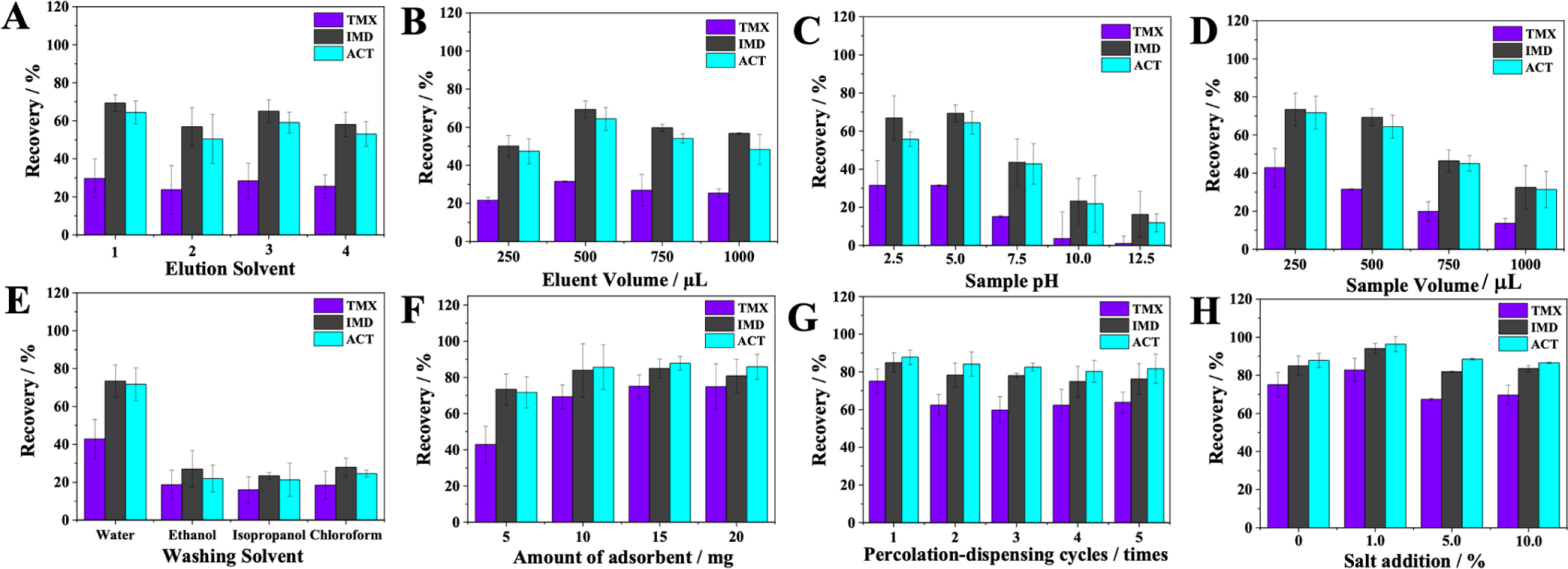
Optimization of sample preparation: (A) Elution solvent: (1) methanol, (2) methanol: acetic acid (9:1, v/v), (3) acetonitrile, (4) dichloromethane. (B) Elution volume; (C) sample pH; (D) sample volume; (E) washing solvent; (F) amount of adsorbent; (G) percolation–dispensing cycles; (H) salt addition.

The recoveries of each insecticide in relation to the type of eluent are shown in Figure 4A. Among the solvents evaluated, the best performance was exhibited by methanol, as it contributed to a greater recovery of all analytes. Due to the moderately polar nature of neonicotinoids, it is important that the elution solvent has a high eluotropic value (ε°).^31^ Among the solvents tested, methanol has the highest ε° (0.73), for this reason, it was able to break the interactions of the analytes with the solid phase more efficiently than the other eluents.

#### 3.2.2 Eluent Volume

The eluent volume was optimized in order to minimize solvent consumption without compromising the extraction efficiency. As shown in Figure 4B, 500 μL of eluent was sufficient to promote the disruption of interactions between the analytes and the adsorbent, consequently leading to greater recovery of all insecticides.

#### 3.2.3 Sample pH

Figure 4C shows that at pH 2.5 and 5.0, the recovery of all analytes was favored; however, at pH 5.0, lower RSD% was obtained for TMX and IMD, in addition to a slight improvement in the recovery of ACT. As the pH of the medium increases, recovery is impaired for all analytes because the pH affected the solubility of the other constituents of the sample that interfered with the adsorption process. Experimentally, the change in color of the sample solution was observed with increasing pH. Therefore, pH 5.0 was taken as the most suitable for use in this sample preparation. It is important to highlight that at pH 5.0, Ag-MPS@MIP has a predominance of negative surface charges (pH_PZC_ = 4.31). From the evaluation of the distribution of electronic microspecies as a function of pH, it can be seen that at pH values lower than 5, the IMD presents a predominance of microspecies in the protonated form; thus, the electrostatic attraction between the material and the molecule contributed to the better adsorption performance and recovery at this pH.^32^ At pH 5.0, TMX and ACT also benefited from recovery despite having a predominance of electrically neutral microspecies. However, it is known that a decrease in pH increases the concentration of neutral species in acidic molecules, as these two neonicotinoids are acidic in nature, the decrease in pH contributed to greater availability of these analytes in the medium and consequently to greater quantity extracted.^31^ Furthermore, it is possible to predict that London dispersion-type forces may have been responsible for the interaction between TMX and ACT and the adsorbent, as it is an interaction of a weak electrostatic nature; this may have favored the desorption of the analytes by the eluent, resulting in greater recoveries.

#### 3.2.4 Sample Volume

As shown in Figure 4D, 250 μL was the volume in which the proportion between the adsorbent and adsorbate was ideal, providing greater extraction efficiency. Larger volumes disfavored adsorption due to the saturation of adsorption sites caused by the greater mass quantity of the analytes and/or interferents.

#### 3.2.5 Washing Solvent

The recovery of the analytes after extraction using four different washing solvents was evaluated. Figure 4E shows that ultrapure water was the solvent that managed to carry out the cleanup without harming the recovery of neonicotinoids. The other solvents were too strong to be used in this stage, since in addition to the interferents they also eluted a considerable part of the analytes, which can be evidenced by the lower recovery obtained using these solvents. Based on these results and taking into account that normally the ideal washing solvent should be similar in nature to the sample solvent, ultrapure water was chosen.^31^

#### 3.2.6 Material Amount

Until now, in all optimized parameters, a fixed amount (5 mg) of Ag-MPS@MIP was used; however, it was necessary to evaluate whether the use of larger amounts would influence the recovery of neonicotinoids. From Figure 4F, it can be seen that the maximum recovery capacity was obtained with 15 mg of adsorbent, an amount that is still much lower compared to conventional SPE.

#### 3.2.7 Percolation–Dispensing Cycles

Better performance of the extraction technique can be achieved by carrying out more than one adsorption cycle, i.e., by percolating an aliquot of the sample repeatedly to improve the adsorption capacity of the material. However, as shown in Figure 4G, only one cycle was sufficient to promote the adsorption of the analytes, and the use of more percolation–dispense cycles did not add efficiency to the extraction. This result was considered positive, as it adds speed to the sample preparation process.

#### 3.2.8 Salting-Out Effect

The salting-out effect (ionic strength) consists of adding a certain concentration of salt, usually sodium chloride, to the sample solution. This feature is widely used in liquid–liquid extraction to increase the availability of analytes in the organic phase. In PT-SPE, the salting-out effect can be useful to improve the adsorption of more polar compounds.^33^ Thus, the influence of adding different concentrations of salt on the recovery of the insecticides was verified. As shown in Figure 4H, the addition of 1% NaCl favored the adsorption of neonicotinoids and contributed to improve the recoveries.

### 3.3 Adsorbent Reuse

**Figure 5:**
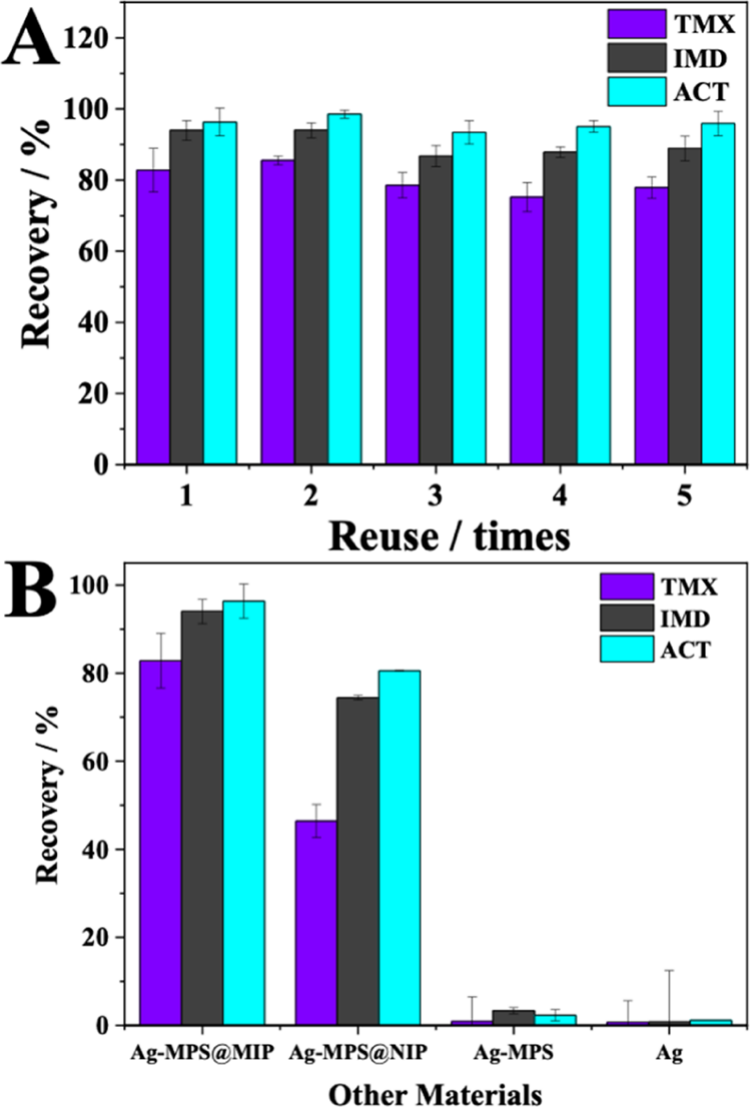
(A) Reuse of the material and (B) comparison of the application of Ag-MPS@MIP in sample preparation with Ag-MPS@NIP, Ag-MPS, and Ag.

According to Figure 5A, the recovery percentage remained constant when the material was used for the second time to extract neonicotinoids under the optimized conditions. Recovery showed a slight drop after the third extraction but maintained a high recovery rate when the material was used five times. It can be said that the total adsorbent capacity of Ag-MPS@MIP is maintained after reuse, which is why it can be used up to five times without significant loss of extraction efficiency.

### 3.4 Comparison of Recoveries of Ag-MPS@MIP with Ag-MPS@MIP, Ag-MPS, and Ag

Figure 5B shows that the best extraction profile was achieved using an adsorbent. The neonicotinoid-selective cavities predetermined during the synthesis contributed to excellent performance in the extraction of these insecticides. However, significant recoveries, mainly for IMD and ACT, were also achieved using Ag-MPS@NIP, which leads to the assumption that the intrinsic characteristics of the core–shell structure, such as greater binding capacity and facilitated mass transfer, certainly contributed to the excellent adsorbent capacity of this material, despite being nonselective. Pure and functionalized silver nanoparticles showed insignificant recovery, which was expected due to the nonadsorbent characteristic of silver.

The high extraction efficiency of neonicotinoids in coconut water was achieved through the optimization of sample preparation using Ag-MPS@MIP as an adsorbent in PT-SPE. Recoveries/RSD% of 82.80 ± 6.16% were obtained for TMX, 94.01 ± 2.75% for IMD and 96.36 ± 3.86% for ACT. All evaluated parameters as well as optimized conditions are summarized in Table S2.

### 3.5 Imprinting Effect

**Figure 6:**
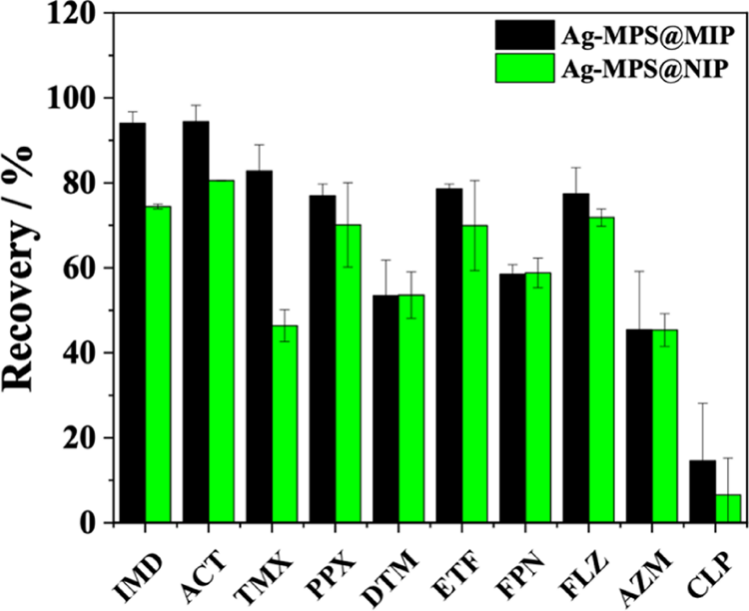
Selectivity test: comparative study of recoveries using Ag-MPS@MIP and Ag-MPS@NIP in the extraction of pyriproxyfen (PPX), deltamethrin (DTM), etofenprox (ETF), fipronil (FPN), fluazuron (FLZ), azamethiphos (AZM), and chlorpyrifos (CLP).

Table S3 shows the experimental results for the selectivity of Ag-MPS@MIP. Distribution coefficient (*K*_d_) measures the affinity of the analyte for the adsorbent; therefore, the higher the *K*_d_ value, the greater the tendency of this molecule to interact with the material’s adsorption sites. Among the analytes studied, IMD presented the highest *K*_d_, followed by ACT and TMX, signaling that when in solution, these analytes move toward Ag-MPS@MIP more easily than the other pesticides evaluated. Compared with Ag-MPS@NIP, neonicotinoids had lower *K*_d_ values, suggesting that the presence of the template in the synthesis of Ag-MPS@MIP positively influences the adsorption of these insecticides. The selectivity coefficient (*K*) measures the affinity of the material for the template with respect to other analytes. It is observed that ACT and TMX presented the lowest *K* values, suggesting that Ag-MPS@MIP can molecularly recognize analytes structurally similar to a template. TMX presented the highest difference in extraction recovery between Ag-MPS@MIP and Ag-MPS@NIP (Figure 6). Finally, the relative selectivity coefficient (*K′*) evaluates the chemical printing process on the material, where *K′* ≥ 1 indicates that the imprinting effect was positive. Evaluating the values of *K*_d_, *K*, and *K′*, it is possible to say that the Ag-MPS@MIP imprinting process was successful since cavities selective were formed during polymerization. A comparative study of recoveries using Ag-MPS@MIP and Ag-MPS@NIP in the extraction of all analytes involved in the selectivity test was also carried out. As can be seen in Figure 6, the extraction efficiency using Ag-MPS@MIP as an adsorbent was superior for neonicotinoids, with recovery close to 100% for IMD. However, Ag-MPS@NIP exhibited excellent performance in the nonselective adsorption of different pesticides, mainly for pyriproxyfen, etofenprox, and fluazuron (Figure 6). These results, in general, demonstrate the existence of molecular recognition sites in the Ag-MPS@MIP, but also show that polymerization on the silver surface contributed to high adsorbent capacity, both selective and nonselective.

### 3.6 Development of HPLC/UV Method

**Figure 7:**
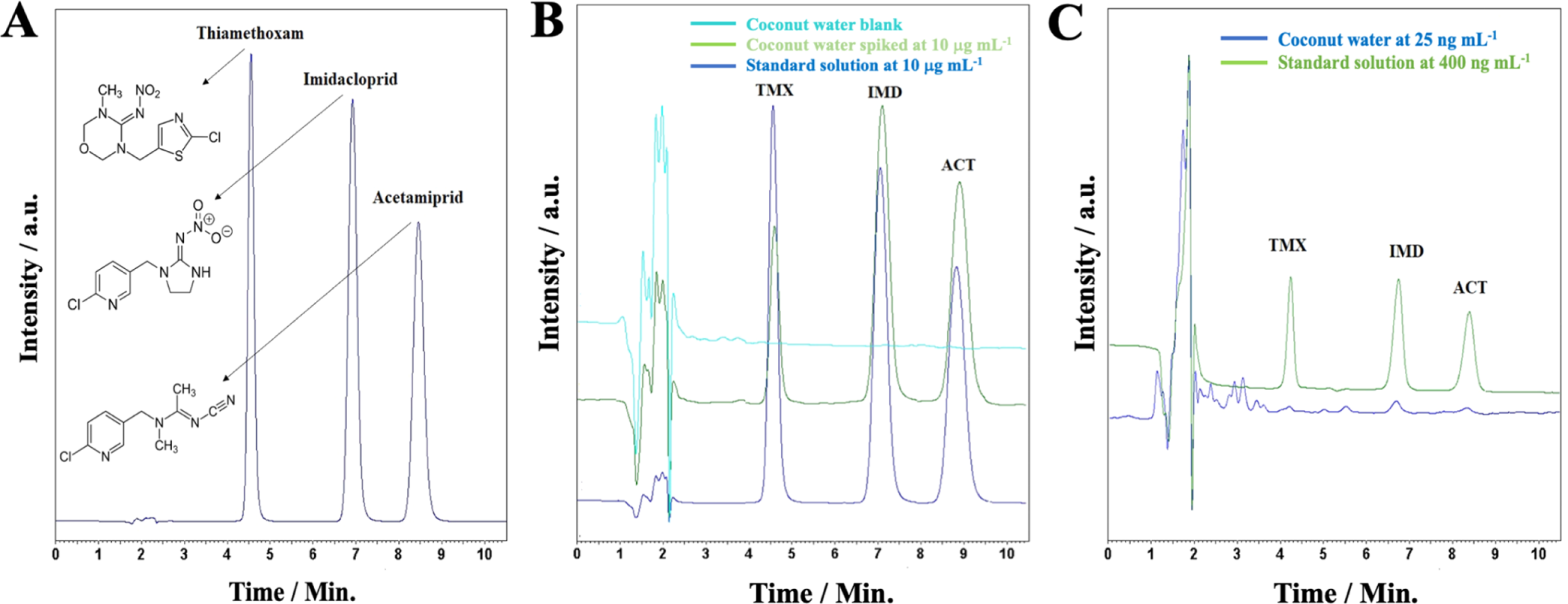
(A) Chromatogram referring to optimal conditions of the separation of the thiamethoxam, imidacloprid, and acetomiprid at 10 μg mL^–1^. (B) Chromatograms of the selectivity study of the neonicotinoids in coconut water sample. (C) Chromatograms referring to the application of the method: the standard solution at 800 ng mL^–1^ (green line) and concentration of 25 ng mL^–1^ (blue line). Conditions: Phenomenex C18 column (150 mm × 4.6 mm, 5 μm), mobile phase composed of ultrapure water acidified with 0.3% phosphoric acid: acetonitrile (78:22, v/v), temperature at 25 °C, flow rate at 1.0 mL min^–1^, injection volume of 10 μL, and λ = 260.

After successive optimizations of the composition of the mobile and stationary phases, it was possible to achieve an optimum separation condition, as shown in Figure 7A. The system suitability tests showed that the retention factor for all of the analytes was within the range of 1–5, which is considered ideal for fast and reliable analytical separation. The repeatability of the analytical responses for the same concentration level (RSD%/*n* = 5) was less than 2%, indicating the accuracy of the system. The values of the separation factor (α > 1), resolution (*R*_s_ ≥ 2.0), and asymmetry factor (*A*_f_ ≈ 1) confirmed that the chromatographic system was able to chemically distinguish the components and that the separation of the analytes was complete. In addition, the peaks showed theoretical plates above 2000 for all of the insecticides, indicating acceptable efficiency.

### 3.7 Method Validation

Figure 7B highlights the selectivity of the method by superimposing the chromatograms of neonicotinoid standards with the chromatogram of pool blank (coconut water samples without analytes and extracted by PT-SPE) and enriched with the analytes and extracted by PT-SPE. It can be seen that in the retention times of the analytes, there is no presence of any signal that could coelute with the peaks or compromise the clear distinction of the pesticides. Table 2 shows the linearity, LOD, and LOQ results of the method. Linearity was evaluated in the range of 15–4000 ng mL^–1^, whose linear regression equations presented correlation coefficients above 0.99 for all analytes;^25,26^ furthermore, the RSD% of the slope of each curve was less than 15%. The *F* values calculated by ANOVA for all analytical curves were lower than *F*_critical_ = 2.93, demonstrating that there is no lack of fit in the linear regression model used. Furthermore, the *p*-values calculated from the *F* test were higher than the significance level (α = 0.05) for all curves, which confirms that the model used was adequate and shows that the instrumental responses varied linearly with the concentrations in the range studied. The LOQ (*n* = 5) of 15 ng mL^–1^ was used as the first point of the analytical curve and presented acceptable precision and accuracy for all neonicotinoids studied.

**Table 2:** Linearity, LOD, and LOQ for the Analytical Method for Determination of Neonicotinoids in Coconut Water Samples

	TMX	IMD	ACT
linear equation^a^	*y* = 877949*x* + 65383	*y* = 1330450*x* + 155928	*y* = 1340464*x* + 72813
*r*^b^	0.9967	0.9958	0.9975
range/ng mL^–1^	15–4000	15–4000	15–4000
RSD%^c^	5.29	2.40	0.69
ANOVA–Lack of fit			
*F-*value^d^	1.81	1.78	0.95
*p-*value^e^	0.259	0.199	0.463
LOD/LOQ			
LOD/ng mL^–1^	5	5	5
LOQ/ng mL^–1^	15	15	15
RSD%^f^	15.59	15.10	12.18
RE%^g^	–5.94	–0.78	–15.76

a Linear equation: referring to the calibration curves determined in triplicate (*n* = 3) for concentrations of 15, 800, 1600, 2400, 3200, 4000 ng mL^–1^; *y* = *ax* + *b*, where *y* is the peak area of the analyte, *a* is the angular coefficient, *b* is the linear coefficient, and *x* is the solution concentration measured in ng mL^–1^.

b *r*: Linear correlation coefficient; RSD%.

c Relative standard deviation of slope of the calibration curve.

d *F*: *F*_calculated_ < *F*_critical_ = 2.93.

e *p*: *p*-value > 0.05: lack of adjustment is not statistically significant; RSD%.

f Relative standard deviation for LOQ (*n* = 5); RE%.

g Relative error for LOQ (*n* = 5).

Table S4 shows the results of the precision and accuracy tests. The RSD%, RE%, and *p*-values for intraday and interday precision and accuracy were acceptable. From the results shown in Table S5, the RSD% values were less than 15%, in both short- and long-term stability. The results of the Student’s *t* test, whose *p-*values were equal to or greater than the significance level (α = 0.05), demonstrated that there was no significant difference between the analytical responses of samples subjected to stability conditions and freshly prepared samples.

### 3.8 Method Application

The method was applied to the analysis of some real samples. However, none of the insecticides studied was found in the samples evaluated. Therefore, the addition of the standard was carried out at concentrations slightly above the LOQ of the method. In Table S6, a comparison is made between the nominal concentration and the actual concentration obtained, as well as the RE% values, all below 15%. Furthermore, Figure 7C shows the superimposed chromatograms of the method application at 25 ng mL^–1^ and the standard solution at 800 ng mL^–1^, confirming that the peaks could be detected and quantified clearly and selectively. It is important to mention that there are no maximum residue levels (MRLs) for coconut water, but there are for the coconut (fruit). In February 2023, the European Commission published Regulation 2023/334 (Commission Regulation—EU No. 334/2023)^34^ and a press release (European Commission—Daily News 02/02/2023: Commission adopts stringent residue limits for pesticides to protect pollinators)^35^ with rules that reduce the authorized Maximum Residue Limits (MRLs) for two neonicotinoids, TMX and clothianidin, to the lowest level that can be measured with the latest available technology. These limits will apply to all food and feed produced and imported into the EU. In Brazil, the MRLs for IMD and ACT in coconut are 0.04 and 0.1 mg kg^–1^, respectively.^36,37^ Furthermore, the MRLs for IMD, TMX, and ACT are higher than the LOQs found in this method, which makes this method consistently applicable and capable of being extended to other matrices.

### 3.9 Comparison with Methods Reported in the Literature

Several studies aimed at determining neonicotinoids in different matrices have been described in the literature in recent years.^38−61^ A summary of these studies is presented in Table S7. It is possible to note that in most of these publications, sample preparation was based on SPE, LLE, and QuEChERS, using liquid chromatography as an instrumental technique. This technique was predominantly used associated with different detection modes and with gradient elution mode most of the time. However, the method developed in this work used isocratic elution, which contributed to the simplicity and speed of the chromatographic analysis and managed to obtain good LOD and LOQ values using UV detection. Furthermore, recoveries close to and even better than those in other studies that determined the same analytes in different matrices were obtained. It is important to highlight that some studies cited analyzed other neonicotinoids simultaneously; however, only the insecticides studied were mentioned in this comparison. However, no study was reported in the literature that used PT-SPE associated with Ag-MPS@MIP to extract neonicotinoids from coconut water samples.

## 4 Conclusions

A new method to determine the three neonicotinoid pesticides in coconut water was developed. For this, an adsorbent with a core–shell structure was used in an unprecedented way using silver nanoparticles modified with γ-MPS as the core and a methacrylic acid-based MIP (Ag-MPS@MIP) as its shell. This material was properly characterized by different instrumental techniques, which brought important information about the physical and chemical characteristics of Ag-MPS@MIP and the synthetic precursor materials, among them are the formation of silver on a nanometric scale, high thermal stability, good adhesion of the polymer to the core, hydrophobic surface, and high surface area. The association of this adsorbent with the PT-SPE provided excellent recoveries for TMX (82.80 ± 6.16%), IMD (94.01 ± 2.75%), and ACT (96.36 ± 3.86%), in addition to high reuse capacity and selectivity. This excellent result is largely due to the greater binding kinetics and lower resistance to mass transfer intrinsic to the core–shell structure but also to the excellent adsorbent and selective capacity of MIP. The method for separating neonicotinoids in isocratic mode showed compliance with the chromatographic system, speed, ease, and efficiency. After validation, the method was satisfactorily applied to real coconut water samples.

## Supporting Information Available

Additional results regarding tables containing estimation of the elemental composition of materials synthesized by EDS; optimized conditions for PT-SPE; optimized conditions for PT-SPE; precision and accuracy of the analytical method for determination of neonicotinoids in coconut water samples; precision and accuracy of the analytical method for determination of neonicotinoids in coconut water samples; application of the method for determining neonicotinoids in coconut water samples; and analytical methods described in the literature for determination of TMX, IMD, and ACT in different matrices (PDF)

## References

[ref1] Kurwadkar S.; Evans A. Neonicotinoids: Systemic Insecticides and Systematic Failure. Bull. Environ. Contam. Toxicol. 2016, 97, 745–748. 10.1007/s00128-016-1968-3. 27872970

[ref2] Maienfisch P.; Huerlimann H.; Rindlisbacher A.; Gsell L.; Dettwiler H.; Haettenschwiler J.; Sieger E.; Walti M. The discovery of thiamethoxam: a second-generation neonicotinoid. Pest Manage. Sci. 2001, 57, 165–176. 10.1002/1526-4998(200102)57:2<165::AID-PS289>3.0.CO;2-G. 11455647

[ref3] Klingelhöfer D.; Braun M.; Brüggmann D.; Groneberg D. A. Neonicotinoids: A critical assessment of the global research landscape of the most extensively used insecticide. Environ. Res. 2022, 213, 113727 10.1016/j.envres.2022.113727. 35732199

[ref4] Thompson D. A.; Lehmler H. J.; Kolpin D. W.; Hladik M. L.; Vargo J. D.; Schilling K. E.; LeFevre G. H.; Peeples T. L.; Poch M. C.; LaDuca L. E.; Cwiertny D. M.; Field R. W. A critical review on the potential impacts of neonicotinoid insecticide use: current knowledge of environmental fate, toxicity, and implications for human health. Environ. Sci.: Processes Impacts 2020, 22, 1315–1346. 10.1039/C9EM00586B. PMC11755762 32267911

[ref5] Chen M.; Tao L.; McLean J.; Lu C. Quantitative analysis of neonicotinoid insecticide residues in foods: implication for dietary exposures. J. Agric. Food Chem. 2014, 62, 6082–6090. 10.1021/jf501397m. 24933495 PMC4081123

[ref6] Sun H.; Feng J.; Han S.; Ji X.; Li C.; Feng J.; Sun M. Recent advances in micro- and nanomaterial-based adsorbents for pipette-tip solid-phase extraction. Microchim. Acta 2021, 188, 189 10.1007/s00604-021-04806-0. 33991231

[ref7] Shi Z.; Li Q.; Xu D.; Huai Q.; Zhang H. Graphene-based pipette tip solid-phase extraction with ultra-high performance liquid chromatography and tandem mass spectrometry for the analysis of carbamate pesticide residues in fruit juice. J. Sep. Sci. 2016, 39, 4391–4397. 10.1002/jssc.201600498. 27647795

[ref8] Su Y.; Wang S.; Zhang N.; Cui P.; Gao Y.; Bao T. Zr-MOF modified cotton fiber for pipette tip solid-phase extraction of four phenoxy herbicides in complex samples. Ecotoxicol. Environ. Saf. 2020, 201, 110764 10.1016/j.ecoenv.2020.110764. 32480162

[ref9] Wu X.; Shen S.; Yan H.; Yuan Y.; Chen X. Efficient enrichment and analysis of atrazine and its degradation products in Chinese Yam using accelerated solvent extraction and pipette tip solid-phase extraction followed by UPLC–DAD. Food Chem. 2021, 337, 127752 10.1016/j.foodchem.2020.127752. 32777573

[ref10] Yang C.; Lv T.; Yan H.; Wu G.; Li H. Glyoxal–urea–formaldehyde molecularly imprinted resin as pipette tip solid-phase extraction adsorbent for selective screening of organochlorine pesticides in spinach. J. Agric. Food Chem. 2015, 63, 9650–9656. 10.1021/acs.jafc.5b02762. 26449689

[ref11] Ye T.; Liu A.; Bai L.; Yuan M.; Cao H.; Yu J.; Yuan R.; Xu X.; Yuan H.; Xu F. Core–satellite surface imprinting polymer-based pipette tip solid-phase extraction for the colorimetric determination of pyrethroid metabolite. Microchim. Acta 2020, 412, 187 10.1007/s00604-020-04394-5. 32601994

[ref12] Dinali L. A. F.; Oliveira H. L.; Teixeira L. S.; Borges W. S.; Borges K. B. Mesoporous molecularly imprinted polymer core@shell hybrid sílica nanoparticles as adsorbent in microextraction by packed sorbent for multiresidue determination of pesticides in apple juice. Food Chem. 2021, 345, 128745 10.1016/j.foodchem.2020.128745. 33302105

[ref13] Villa C. C.; Sánches L. T.; Valencia G. A.; Ahmed S. Molecularly imprinted polymers for food applications: A review. Trends Food Sci. Technol. 2021, 111, 642–669. 10.1016/j.tifs.2021.03.003.

[ref14] Hayes R.; Ahmed A.; Edge T.; Zhang H. Core–shell particles: Preparation, fundamentals and applications in high performance liquid chromatography. J. Chromatogr. A 2014, 1357, 36–52. 10.1016/j.chroma.2014.05.010. 24856904

[ref15] Bhogal S.; Kaur K.; Malik A. K.; Sonne C.; Lee S. S.; Kim K.-H. Core-shell structured molecularly imprinted materials for sensing applications. Trends Anal. Chem. 2020, 133, 116043 10.1016/j.trac.2020.116043.

[ref16] Aguilar-García D.; Ochoa-Terán A.; Paraguay-Delgado F.; Díaz-García M. E.; Pina-Luis G. Water-compatible core–shell Ag@SiO_2_ molecularly imprinted particles for the controlled release of tetracycline. J. Mater. Sci. 2016, 51, 5651–5663. 10.1007/s10853-016-9867-x.

[ref17] Singh R.; Bhateria R. Core–shell nanostructures: a simplest two-component system with enhanced properties and multiple applications. Environ. Geochem. Health 2021, 43, 2459–2482. 10.1007/s10653-020-00766-1. 33161517

[ref18] Ibrahim W. A. W.; Sutirman Z. A.; Qaderi J.; Bakar K. A.; Basir S. H. M.; Aouissi I. E. A review on applications of gold and silver-based sorbents in solid phase extraction and solid phase microextraction. Malaysian J. Anal. Sci. 2020, 24, 464–483.

[ref19] Cheshari E. C.; Ren X.; Li X. Core–shell Ag-dual template molecularly imprinted composite for detection of carbamate pesticide residues. Chem. Pap. 2021, 75, 3679–3693. 10.1007/s11696-021-01594-y.

[ref20] Ren X.; Cheshari E. C.; Qi J.; Li X. Silver microspheres coated with a molecularly imprinted polymer as a SERS substrate for sensitive detection of bisphenol A. Microchim. Acta 2018, 242, 185 10.1007/s00604-018-2772-z. 29610992

[ref21] Zhang B.; Xu P.; Xie X.; Wei H.; Li Z.; Mack N. H.; Han X.; Xu H.; Wang H.-L. Acid-directed synthesis of SERS-active hierarchical assemblies of silver nanostructures. J. Mater. Chem. 2011, 21, 2495–2501. 10.1039/C0JM02837A.

[ref22] Guo Y.; Kang L.; Chen S.; Li X. High performance surface-enhanced Raman scattering from molecular imprinting polymer capsulated silver spheres. Phys. Chem. Chem. Phys. 2015, 17, 21343 10.1039/C5CP00206K. 25759203

[ref23] Monshi A.; Foroughi M. R.; Monshi M. R. Modified Scherrer equation to estimate more accurately nano-crystallite size using XRD. World J. Nano Sci. Eng. 2012, 2, 154–160. 10.4236/wjnse.2012.23020.

[ref24] Khan M. N.; Sarwar A. Determination of points of zero charge of natural and treated adsorbents. Surf. Rev. Lett. 2007, 14, 461–469. 10.1142/S0218625X07009517.

[ref25] Guidance document SANTE/11945/2015 rev. 0 on analytical quality control and method validation procedures for pesticides residues analysis in food and feed, 2015. https://www.eurl-pesticides.eu/library/docs/allcrl/AqcGuidance_SANTE_2015_11945.pdf. Accessed on July 3, 2024.

[ref26] Australian Pesticides & Veterinary Medicines Authority (APVMA), Validation of analytical methods for active constituent, agricultural and veterinary chemical products, June 14 2023, https://www.apvma.gov.au/registrations-and-permits/data-requirements/agricultural-data-guidelines/chemistry-manufacture-part-2/validation. Accessed on July 30, 2024.

[ref27] Silva R. C. S.; Santos M. N.; Pires B. C.; Dinali L. A. F.; Suquila F. A. C.; Tarley C. R. T.; Borges K. B. Assessment of surfactants on performance of molecularly imprinted polymer toward adsorption of pharmaceutical. J. Environ. Chem. Eng. 2019, 7, 103037 10.1016/j.jece.2019.103037.

[ref28] Malekzadeh M.; Yeung K. L.; Halali M.; Chang Q. Synthesis of nanostructured Ag@SiO_2_-Penicillin from high purity Ag NPs prepared by electromagnetic levitation melting process. Mater. Sci. Eng., C 2019, 102, 616–622. 10.1016/j.msec.2019.04.083. 31147033

[ref29] Pawar H.; Deshpande N.; Dagade S.; Waghmode S.; Joshi P. N. Green synthesis of silver nanoparticles from purple acid phosphatase apoenzyme isolated from a new source *Limonia acidissima*. J. Exp. Nanosci. 2015, 11, 28–37. 10.1080/17458080.2015.1025300.

[ref30] Dinali L. A. F.; Oliveira H. L.; Teixeira L. S.; Silva A. T. M.; D’Oliveira K. A.; Cuin A.; Borges K. B. Efficient development of a magnetic molecularly imprinted polymer for selective determination of trimethoprim and sulfamethoxazole in milk. Microchem. J. 2020, 154, 104648 10.1016/j.microc.2020.104648.

[ref31] Teixeira R. A.; Dinali L. A. F.; de Oliveira H. L.; da Silva A. T. M.; Borges K. B. Efficient and selective extraction of azamethiphos and chlorpyrifos residues from mineral water and grape samples using magnetic mesoporous molecularly imprinted polymer. Food Chem. 2021, 361, 130116 10.1016/j.foodchem.2021.130116. 34029898

[ref32] Chemicalize, neonicotinoids pKa. https://chemicalize.org/. Accessed in Mar. 2022.

[ref33] Vidal D. F.; Pires B. C.; Borges M. M. C.; Oliveira H. L.; Silva C. F.; Borges K. B. Magnetic solid-phase extraction based on restricted-access molecularly imprinted polymers for ultrarapid determination of ractopamine residues from milk and meat samples by capillary electrophoresis. J. Chromatogr. A 2024, 1720, 464809 10.1016/j.chroma.2024.464809. 38490141

[ref34] Commission Regulation (EU) 2023/334 of 2 February 2023 amending Annexes II and V to Regulation (EC) No 396/2005 of the European Parliament and of the Council as regards maximum residue levels for clothianidin and thiamethoxam in or on certain products (Text with EEA relevance) https://eur-lex.europa.eu/eli/reg/2023/334/oj. Accessed on July 3, 2024.

[ref35] European Commission, 2023. Commission adopts stringent residue limits for pesticides to protect pollinators, https://ec.europa.eu/commission/presscorner/detail/en/mex_23_543. Accessed on July 3, 2024.

[ref36] Agência Nacional de Vigilância Sanitária (ANVISA), acetamiprido, https://www.gov.br/anvisa/pt-br/setorregulado/regularizacao/agrotoxicos/monografias/monografias-autorizadas/a/4150json-file-1. Accessed on July 3, 2024.

[ref37] Agência Nacional de Vigilância Sanitária (ANVISA), imidacloprid, https://www.gov.br/anvisa/pt-br/setorregulado/regularizacao/agrotoxicos/monografias/monografias-autorizadas/g-h-i/4400json-file-1. Accessed on July 3, 2024.

[ref38] Obana H.; Okihashi M.; Akutsu K.; Kitagawa Y.; Hori S. Determination of neonicotinoid pesticide residues in vegetables and fruits with solid phase extraction and liquid chromatography mass spectrometry. J. Agric. Food Chem. 2003, 51, 2501–2505. 10.1021/jf0261102. 12696927

[ref39] Fidente P.; Seccia S.; Vanni F.; Morrica P. Analysis of nicotinoid insecticides residues in honey by solid matrix partition clean-up and liquid chromatography–electrospray mass spectrometry. J. Chromatogr. A 2005, 1094, 175–178. 10.1016/j.chroma.2005.09.012. 16257305

[ref40] Muccio A. D.; Fidente P.; Barbini D. A.; Dommarco R.; Seccia S.; Morrica P. Application of solid-phase extraction and liquid chromatography–mass spectrometry to the determination of neonicotinoid pesticide residues in fruit and vegetables. J. Chromatogr. A 2006, 1108, 1–6. 10.1016/j.chroma.2005.12.111. 16448655

[ref41] Guzsvány V.; Madžgalj A.; Trebše P.; Gaál F.; Franko M. Determination of selected neonicotinoid insecticides by liquid chromatography with thermal lens spectrometric detection. Environ. Chem. Lett. 2007, 5, 203–208. 10.1007/s10311-007-0102-5.

[ref42] Seccia S.; Fidente P.; Montesano D.; Morrica P. Determination of neonicotinoid insecticides residues in bovine milk samples by solid-phase extraction clean-up and liquid chromatography with diode-array detection. J. Chromatogr. A 2008, 1214, 115–120. 10.1016/j.chroma.2008.10.088. 19004450

[ref43] Xie W.; Han C.; Qian Y.; Ding H.; Chen X.; Xi J. Determination of neonicotinoid pesticides residues in agricultural samples by solid-phase extraction combined with liquid chromatography–tandem mass spectrometry. J. Chromatogr. A 2011, 1218, 4426–4433. 10.1016/j.chroma.2011.05.026. 21641601

[ref44] Zhang F.; Li Y.; Yu C.; Pan C. Determination of six neonicotinoid insecticides residues in spinach, cucumber, apple and pomelo by QuEChERS method and LC-MS/MS. Bull. Environ. Contam. Toxicol. 2012, 88, 885–890. 10.1007/s00128-012-0579-x. 22398693

[ref45] Chen L.; Li B. Determination of imidacloprid in rice by molecularly imprinted-matrix solid-phase dispersion with liquid chromatography tandem mass spectrometry. J. Chromatogr. B 2012, 897, 32–36. 10.1016/j.jchromb.2012.04.004. 22534657

[ref46] Xiao Z.; Yang Y.; Li Y.; Fan X.; Ding S. Determination of neonicotinoid insecticides residues in eels using subcritical water extraction and ultra-performance liquid chromatography–tandem mass spectrometry. Anal. Chim. Acta 2013, 777, 32–40. 10.1016/j.aca.2013.03.026. 23622962

[ref47] Yáñez K. P.; Bernal J. L.; Nozal M. J.; Martín M. T.; Bernal J. Determination of seven neonicotinoid insecticides in beeswax by liquid chromatography coupled to electrospray-mass spectrometry using a fused-core column. J. Chromatogr. A 2013, 1285, 110–117. 10.1016/j.chroma.2013.02.032. 23473513

[ref48] Campillo N.; Viñas P.; Férez-Melgarejo G.; Hernández-Córdoba M. Liquid chromatography with diode array detection and tandem mass spectrometry for the determination of neonicotinoid insecticides in honey samples using dispersive liquid–liquid microextraction. J. Agric. Food Chem. 2013, 61, 4799–4805. 10.1021/jf400669b. 23668600

[ref49] Jovanov P.; Guzsvány V.; Franko M.; Lazić S.; Sakač M.; Milovanović I.; Nedeljković N. Development of multiresidue DLLME and QuEChERS based LC–MS/MS method for determination of selected neonicotinoid insecticides in honey liqueur. Food Res. Int. 2014, 55, 11–19. 10.1016/j.foodres.2013.10.031.

[ref50] Gbylik-Sikorska M.; Sniegocki T.; Posyniak A. Determination of neonicotinoid insecticides and their metabolites inhoney bee and honey by liquid chromatography tandem massspectrometry. J. Chromatogr. B 2015, 990, 132–140. 10.1016/j.jchromb.2015.03.016. 25864015

[ref51] Jovanov P.; Guzsvány V.; Lazic S.; Franko M.; Sakac M.; Saric L.; Kos J. Development of HPLC-DAD method for determination of 4 neonicotinoids in honey. J. Food Compos. Anal. 2015, 40, 106–113. 10.1016/j.jfca.2014.12.021.

[ref52] Jiao W.; Xiao Y.; Qian X.; Tong M.; Hu Y.; Hou R.; Hua R. Optimized combination of dilution and refined QuEChERS to overcome matrix effects of six types of tea for determination eight neonicotinoid insecticides by ultra performance liquid chromatography–electrospray tandem mass spectrometry. Food Chem. 2016, 210, 26–34. 10.1016/j.foodchem.2016.04.097. 27211616

[ref53] Rodrígues-Cabo T.; Casado J.; Rodrígues I.; Ramil M.; Cela R. Selective extraction and determination of neonicotinoid insecticidesin wine by liquid chromatography–tandem mass spectrometry. J. Chromatogr. A 2016, 1460, 9–15. 10.1016/j.chroma.2016.07.004. 27425763

[ref54] Lachat L.; Glauser G. Development and validation of an ultra-sensitive UHPLC–MS/MS method for neonicotinoid analysis in milk. J. Agric. Food Chem. 2018, 66, 8639–8646. 10.1021/acs.jafc.8b03005. 30025459

[ref55] Song S.; Zhang C.; Chen Z.; He F.; Wei J.; Tan H.; Li X. Simultaneous determination of neonicotinoid insecticides and insectgrowth regulators residues in honey using LC–MS/MS with anionexchanger-disposable pipette extraction. J. Chromatogr. A 2018, 1557, 51–61. 10.1016/j.chroma.2018.05.003. 29735281

[ref56] Suganthi A.; Bhuvaneswari K.; Ramya M. Determination of neonicotinoid insecticide residues in sugarcane juice using LCMSMS. Food Chem. 2018, 241, 275–280. 10.1016/j.foodchem.2017.08.098. 28958529

[ref57] Kachangoon R.; Vichapong J.; Burakham R.; Santaladchaiyakit Y.; Srijaranai S. Ultrasonically modified amended-cloud point extraction for simultaneous pre-concentration of neonicotinoid insecticide residues. Molecules 2018, 23, 1165 10.3390/molecules23051165. 29757232 PMC6100087

[ref58] Farooq S.; Nie J.; Cheng Y.; Yan Z.; Bacha S. A. S.; Zhang J.; Nahiyoon R. A.; Hussain Q. Synthesis of core-shell magnetic molecularly imprinted polymer for the selective determination of imidacloprid in apple samples. J. Sep. Sci. 2019, 42, 2455–2465. 10.1002/jssc.201900221. 31070852

[ref59] Yang C.; Ran L.; Xu M.; Ren D.; Li L. *In situ* ionic liquid dispersive liquid–liquid microextraction combined with ultra high-performance liquid chromatography for determination of neonicotinoid insecticides in honey samples. J. Sep. Sci. 2019, 42, 1930–1937. 10.1002/jssc.201801263. 30869190

[ref60] Wang D.; Liu Y.; Xu Z.; Ji Y.; Si X.; Lin T.; Liu H.; Liu Z. Generic imprinted fiber array strategy for high-throughput and ultrasensitive simultaneous determination of multiple neonicotinoids. Food Chem. 2022, 382, 132407 10.1016/j.foodchem.2022.132407. 35152016

[ref61] Waleng N. J.; Selahle S. K.; Mpupa A.; Nomngongo P. N. Development of dispersive solid-phase microextraction coupled with high-pressure liquid chromatography for the preconcentration and determination of the selected neonicotinoid insecticides. J. Anal. Sci. Technol. 2022, 13, 4 10.1186/s40543-021-00311-4.

