# Supplementary material for: Silver Core Coated with Molecularly Imprinted Polymer as Adsorbent in Pipet-Tip Solid Phase Extraction for Neonicotinoids Determination from Coconut Water
Source: ACS Meas Sci Au. 2024 Aug 28;4(5):556–67. doi: 10.1021/acsmeasuresciau.4c00036 (PMC11487786; doi:10.1021/acsmeasuresciau.4c00036)
Supplement: Supplementary file 1 — tg4c00036_si_001.pdf [file tg4c00036_si_001.pdf]

Supplementary material

Silver-core coated with molecularly imprinted polymer as adsorbent in pipette-tip solid phase extraction for neonicotinoids determination from coconut water

Laíse Aparecida Fonseca Dinali, Anny Talita Maria da Silva, and Keyller Bastos Borges*

Departamento de Ciências Naturais, Universidade Federal de São João del-Rei (UFSJ), Campus Dom Bosco, Praça Dom Helvécio 74, Fábricas, 36301-160, São João del-Rei, Minas Gerais, Brazil.

Correspondence:

Prof. Keyller Bastos Borges, Ph.D., Departamento de Ciências Naturais, Universidade Federal de São João del-Rei, Campus Dom Bosco, Praça Dom Helvécio 74, Fábricas, 36301-160, São João del-Rei, Minas Gerais, Brazil. e-mail: keyller@ufsj.edu.br; Phone number: +55 32 3379–5163

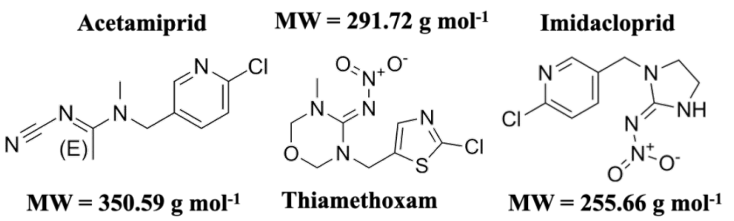

Figure S1. Chemical structure of acetamiprid (ACT), thiamethoxam (TXM) and imidacloprid (IMD).

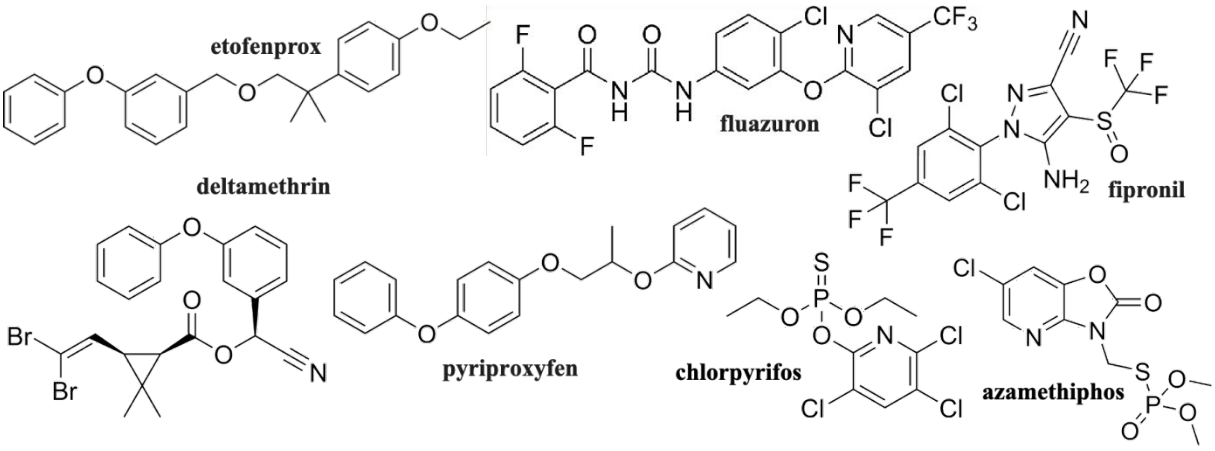

Figure S2. Chemical structures of pyriproxyfen (PPX, MW= 321.37 g mol^{-1}), deltamethrin (DTM, MW= 505.21 g mol^{-1}), etofenprox (ETF, MW= 376.49 g mol^{-1}), fipronil (FPN, MW= 437.15 g mol^{-1}), fluazuron (FLZ, MW= 506.21 g mol^{-1}), azamethiphos (AZM, MW= 324.67 g mol^{-1}) and chlorpyrifos (CLP, MW= 350.49 g mol^{-1}) that were used in the selectivity study.

Table S1. Estimation of the elemental composition of materials synthesized by EDS.

Material	Element (%)				
	Ag	Si	C	O	Impurity [a]
**Ag**	98.9	-	-	-	1.1
**Ag-MPS**	98.2	0.30	0.60	0.90	-
**Ag@MIP**	2.10	0.10	62.4	35.4	-
**Ag@NIP**	2.50	0.10	59.8	37.6	-

[a] other elements identified

Table S2. Optimized conditions for PT-SPE

**Parameters**	**Optimized conditions**
Elution solvent	Methanol
Eluent volume	500 μL
Sample pH	5.0
Sample volume	250 μL
Washing solvent	Ultrapure water
Amount of Ag-MPS@MIP	15 mg
Percolation-dispensing cycles	1 ×
Salt addition	1.0 %
Reuse	5 ×
Enrichment factor	5

The eluates were dried, resuspended in 50 μL of methanol and submitted to chromatographic analysis.

Table S3. Distribution coefficients (*Kd*), selectivity coefficients (*K*) and relative selectivity coefficient (*K'*) for neonicotinoids and interfering.

	IMD	ACT	TMX	PPX	DTM	ETF	FPN	FLZ	AZM	CLP
					***Kd* / mL g^{-1}**					
**Ag-MPS@MIP**	261	215	80.3	60.9	19.2	63.5	23.5	57.1	13.9	2.84
**Ag-MPS@NIP**	48.5	68.9	15.2	39.1	19.2	38.8	23.8	42.5	13.8	1.22
					K					
**Ag-MPS@MIP**	-	1.22	3.26	4.29	13.7	4.11	11.1	4.58	18.8	92.0
**Ag-MPS@NIP**	-	0.70	3.20	1.24	2.52	1.25	2.04	1.14	3.51	41.5
					K'					
	-	1.73	1.02	3.46	5.41	3.29	5.46	4.01	5.37	2.22

IMD, imidacloprid; ACT, acetamiprid; TMX, thiamethoxan; PPX, pyriproxyfen; DTM, deltamethrin; ETF, etofemprox; FPN, fipronil; FLZ, fluazuron; AZM, azamethiphos CLP, chlorpyriphos.

Table S4. Precision and accuracy of the analytical method for determination of neonicotinoids in coconut water samples.

	TMX			IMD			ACT		
**Nominal concentration / ng mL^{-1}**	800	2400	4000	800	2400	4000	800	2400	4000
**Intraday / n[a] = 5**									
**Analyzed concentration / ng mL^{-1}**	850	2330	4030	850	2280	4070	840	2350	4020
**Precision / RSD% [c]**	9.64	5.66	3.55	5.95	5.40	3.76	7.46	5.64	1.92
**Accuracy / RE% [d]**	6.32	-3.08	0.65	6.45	-5.19	1.87	5.55	-2.16	0.53
**Inter-day / n[b] = 2**									
**Analyzed concentration / ng mL^{-1}**	830	2360	4050	850	2330	4060	830	2330	3990
**Precision / RSD% [c]**	2.58	4.93	4.61	0.52	5.26	1.88	0.62	3.07	1.20
**Accuracy / RE% [d]**	3.87	-1.69	1.14	5.66	-2.87	1.60	4.11	-2.95	-0.15
***p*-value[e]**	0.39	0.05	0.09	0.82	0.09	0.42	0.85	0.29	0.34

[a] n = number of replicates; [b] n = number of days; [c] RSD% = relative standard deviation percentage between replicates; [d] RE% = relative error percentage between actual and nominal concentration; [e] *p*-value of *t* test for significance level of 95% ($p > 0.05$).

Table S5. Stability test for neonicotinoids studied in coconut water samples.

Conditions	Nominal Concentration / µg mL^{-1}	TMX		IMD		ACT	
		p-value[*b*]	RSD%[c]	*p*-value[b]	RSD%[c]	*p*-value[b]	RSD%[c]
**24 h at 23±3 °C** [a]	800	0.12	10.9	0.58	11.3	0.85	14.7
	4000	0.41	8.59	0.26	6.25	0.24	5.56
**96 h freeze** [a]	800	0.96	12.6	0.34	11.9	0.55	9.89
	4000	0.16	7.74	0.05	7.37	0.05	7.84

[a] n = 5, number of repetitions; [b] Significance level of $p > 0.05$; [c] RSD%, mean relative standard deviation of five replicates.

Table S6. Application of the method for determining neonicotinoids in coconut water samples.

	Concentration / ng mL^{-1}								
	TMX			IMD			ACT		
**Spiked**	25.0	50.0	100.0	25.0	50.0	100.0	25.0	50.0	100.0
**Found [a]**	21.4	49.0	87.9	29.4	43.7	91.9	23.5	44.0	87.5
**RE% [b]**	-14.6	-1.9	-12.2	0.2	-12.6	-8.1	6.2	-12.0	-12.5

Found [a], concentration obtained from a pool of samples and calculated through analytical curves; RE% [b], relative error (n=3).

Table S7. Analytical methods described in literature for determination of TMX, IMD and ACT in different matrices.

Analytes	Sample	Sample preparation	Instrumental technique	LOD / unit	LOQ /unit	Linear range / unit	Recovery / %	Column/elution mode: G[m]/I[n]	Ref.
TMX IMD ACT	Vegetables and fruits	SPE	LC-MS	0.01–0.02 mg kg^{-1}	0.01–0.02 mg kg^{-1}	10–5000 ng mL^{-1}	70–95	Cadenza-C18 (75 × 4.6 mm, 3 µm). G[m]	(38)
TMX IMD ACT	Honey	SPE	LC-ESI-MS	0.01–0.1 mg kg^{-1}	0.03–0.3 mg kg^{-1}	500–50,000 ng mL^{-1}	76–99	Lichrosphere 100 (5µm). G[m]	(39)
TMX IMD ACT	Vegetables and fruits	SPE	LC-ESI-MS	0.02–0.1 mg kg^{-1}	0.1–0.5 mg kg^{-1}	10–1000 ng mL^{-1}	74.5–105	LiChrospher 100 (5 µm). G[m].	(40)
TMX IMD ACT	Potato and water	LLE	HPLC-TLS	3.2–27 ng mL^{-1}	10–89 ng mL^{-1}	-	78.0–93.0	Pinnacle ODS (250 × 4.6 mm, 5 µm). I[n].	(41)
TMX IMD ACT	Milk	SPE	HPLC-DAD	0.003–0.01 mg kg^{-1}	0.01–0.04 mg kg^{-1}	10–1000 ng mL^{-1}	85.1–99.7	Synergi Hydro-C18 (250 × 4.6 mm, 4 µm). I[n].	(42)
TMX IMD ACT	Agricultural samples	SPE	LC-MS/MS	-	0.01–0.02 mg Kg^{-1}	4–100 ng mL^{-1}	82.1–108.5	Zorbax-C8 (150 × 4.6 mm, 5 µm). G[m]	(43)
TMX IMD ACT	Spinach, cucumber, apple and pomelo	QuEChERS	LC-MS/MS	0.20–0.85 µg kg^{-1}	0.66–2.84 µg kg^{-1}	5–1000 ng mL^{-1}	73.7–103.8	Zorbax-C18 (50 × 2.1 mm, 1.8 µm). I[n].	(44)
IMD	Rice	MSPD[a]	LC-MS/MS	2.4 µg kg^{-1}	8.0 µg kg^{-1}	10–1000 µg kg^{-1}	83.8–92.5	Hypersil ODS-C18 (250 × 4.6 mm, 5 µm). I[n].	(45)
TMX IMD ACT	Eel	SWE[b]	UPLC-MS/MS	0.12–0.36 µg kg^{-1}	0.42–1.12 µg kg^{-1}	0–500 µg kg^{-1}	84.6–102.0	Acquity-C18 (50 × 2.1 mm, 1.7 µm). G[m].	(46)
TMX IMD ACT	Beeswax	SPE	LC-ESI/MS	0.4–2.3 µg kg^{-1}	1.5–7.0 µg kg^{-1}	1.5–1000 µg kg^{-1}	85.0–105.0	Kinetex-C18 (150 × 4.6 mm, 2.6 µm). G[m].	(47)

TMX<br>IMD<br>ACT	Honey	SPE-DLLME	HPLC-APCI-IT-MS/MS	0.02–0.13 µg kg^{-1}	0.07–0.43 µg kg^{-1}	0.1–1000 µg kg^{-1}	90.0–104.0	Spherisorb ODS2 (150 × 4 mm, 5 µm). G[m].	(48)
TMX<br>IMD<br>ACT	Honey liqueur	DLLME[c] QuEChERS	LC-MS/MS	0.5–1.5<br>1.0–2.5<br>ng mL^{-1}	1.0–5.0<br>2.5–10.0<br>ng mL^{-1}	1.0–100<br>2.5–100<br>ng mL^{-1}	69.2–113.4<br>71.8–94.9	Zorbax-C18 (50 x 4.6 mm, 1.8 µm). G[m].	(49)
TMX<br>IMD<br>ACT	Honey	SPE	LC-MS/MS	-	0.1–0.5 µg kg^{-1}	0.1–500 µg kg^{-1}	85.3–112.0	Luna-C18 (100 × 2.0 mm, 3 µm). G[m]	(50)
TMX<br>IMD<br>ACT	Honey	DLLME QuEChERS	HPLC-DAD	1.5–2.5<br>2.0–2.5<br>µg kg^{-1}	5.0–7.5<br>5.0–10<br>µg kg^{-1}	5.0–100 µg kg^{-1}	73.4–118.3<br>73.8–97.3	Zorbax-C18 (50 × 4.6 mm, 1.8 µm G[m]	(51)
TMX<br>IMD<br>ACT	Tea	QuEChERS modificado	LC-ESI-MS/MS	-	0.01–0.05 mg kg^{-1}	0.025–25 ng mL^{-1}	66.3–108.0	Waters HSS T3 (100 × 2.1 mm, 1.8 µm). G[m]	(52)
TMX<br>IMD<br>ACT	Wine	SPE	LC-ESI-MS/MS	-	0.1–0.2 ng mL^{-1}	1–500 ng mL^{-1}	61.0–86.0	Zorbax-C18 (100 × 2 mm, 3.5 µm). G[m]	(53)
TMX<br>IMD<br>ACT	Milk	LLE	UHPLC-MS/MS	-	0.0005–0.005 ng mL^{-1}	0.0005–50 ng mL^{-1}	64–76	Cortecs-C18 (50 × 2.1 mm, 1.6 µm). G[m]	(54)
TMX<br>IMD<br>ACT	Honey	DPX[d]	LC-MS/MS	1.0–5.0 mg kg^{-1}	0.3–1.5 mg kg^{-}	1–1000 ng mL^{-1}	72–104	Zorbax-C18 (100 × 2.1 mm, 3.5 µm). G[m].	(55)
TMX<br>IMD<br>ACT	Sugar cane	QuEChERS	LC-MS/MS	0.7–2.0 µg kg^{-1}	2.0–5.0 µg kg^{-1}	5.0–500 ng mL^{-1}	62.1–129.9	Waters-C18 (250 × 4.8 mm, 5 µm). I[n].	(56)
TMX<br>IMD<br>ACT	Water	CPE	HPLC-UV	0.3–2.0 ng mL^{-1}	1.0–6.0 ng mL^{-1}	5.0–700 ng mL^{-1}	60.1–117.6	Chromolith-C18 endcapped (100 × 4.6 mm). I[n].	(57)
IMD	Apple	MSPE[f]	HPLC-UV	48.0 ng mL^{-1}	146 ng mL^{-1}	50–1000 ng mL^{-1}	77.7–96.6	Symmetry-C18 (250 × 4.6 mm, 5 µm. I[n].	(58)

IMD<br>ACT	Honey	DLLME	UHPLC-UV	0.30–0.62<br>ng mL^{-1}	1.20–2.50<br>ng mL^{-1}	1.2–5000<br>ng mL^{-1}	81.0–103.4	Shim-pack XR-ODS II-C18 (75 × 2 mm, 2.2 µm). G^m	(59)
TMX<br>IMD<br>ACT	Tea and honey	SPME	HPLC-DAD	0.03–0.58<br>ng mL^{-1}	0.09–1.93<br>ng mL^{-1}	1–200<br>ng mL^{-1}	87.0–116.9	J&K Scientific-C18 (250 × 4.6 mm, 5 µm). I^n.	(60)
IMD<br>ACT	Waste and river water	d -MSPME	HPLC-DAD	0.41–0.82<br>ng mL^{-1}	1.4–2.7<br>ng L^{-1}	1.4–700<br>ng mL^{-1}	86.7–99.2	Zorbax-C18 (150 × 4.6 mm, 3.5 µm). I^n.	(61)
TMX<br>IMD<br>ACT	Coconut water	PT-SPE	HPLC-UV	5.0<br>ng mL^{-1}	15<br>ng mL^{-1}	15–4000<br>ng mL^{-1}	82.8–96.4	Phenomenex C18 (150 × 4.6 mm, 5 µm). I^n.	This work

MSPD, matrix solid-phase dispersion; SWE, subcritical water extraction; DLLME, Dispersive liquid-liquid microextraction; DPX, disposable pipette extraction; CPE, cloud point extraction; MSPE, magnetic solid phase extraction; SPME, solid phase microextraction; d -MSPME. dispersive magnetic solid-phase microextraction; LC-ESI-MS/MS, LC–electrospray ionization mass spectrometry; HPLC/TLS, LC–thermal lens spectrometry; HPLC/APCI-IT-MS/MS, LC–atmospheric pressure chemical ionization–ion trap–tandem mass spectrometry; G^m: gradient elution; I^n: Isocratic elution.
